# Supplementary material for: Bimetallic AgPt Nanoalloys as an Electrocatalyst for Ethanol Oxidation Reaction: Synthesis, Structural Analysis, and Electro-Catalytic Activity
Source: Nanomaterials (Basel). 2023 Apr 18;13(8):1396. doi: 10.3390/nano13081396 (PMC10145194; doi:10.3390/nano13081396)
Supplement: Supplementary file 1 [file nanomaterials-13-01396-s001.zip › nanomaterials-2332310-supplementary.pdf]

Supplementary Materials

Bimetallic AgPt Nanoalloys as an Electrocatalyst for Ethanol Oxidation Reaction: Synthesis, Structural Analysis, and Electro-Catalytic Activity

According to Sigma-Aldrich provender information, the technical specifications of the Pt/C commercial electrocatalyst (PRD.0.ZQ5.10000029756) are 20% wt. of Pt loading and crystallite size ≤ 5 nm confirmed by TGA and XRD techniques, respectively. On the other hand, the particle distribution was determined by the SEM-SE technique (Figure S1). The micrograph shows particles well dispersed and particles agglomeration on different zones of the carbon support.

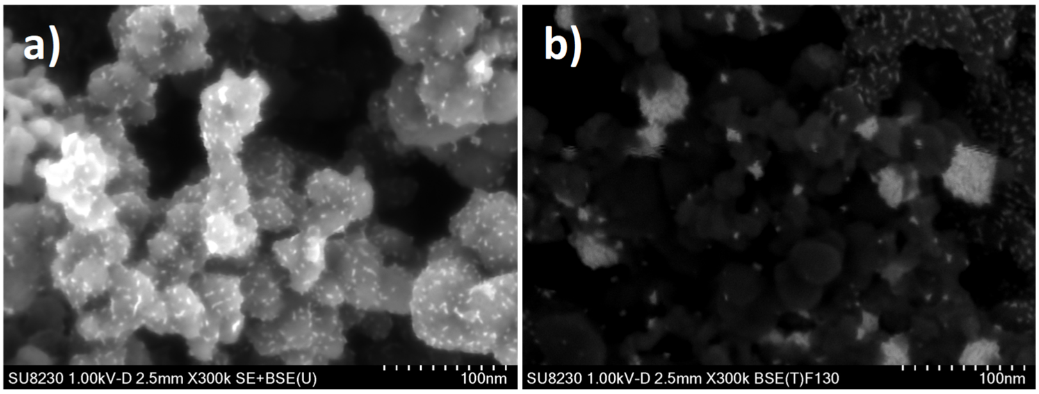

Figure S1. Low-magnification SEM micrographs of Pt/C commercial electrocatalyst purchased from Sigma-Aldrich; a) Secondary electrons image and b) Backscattered electron image.

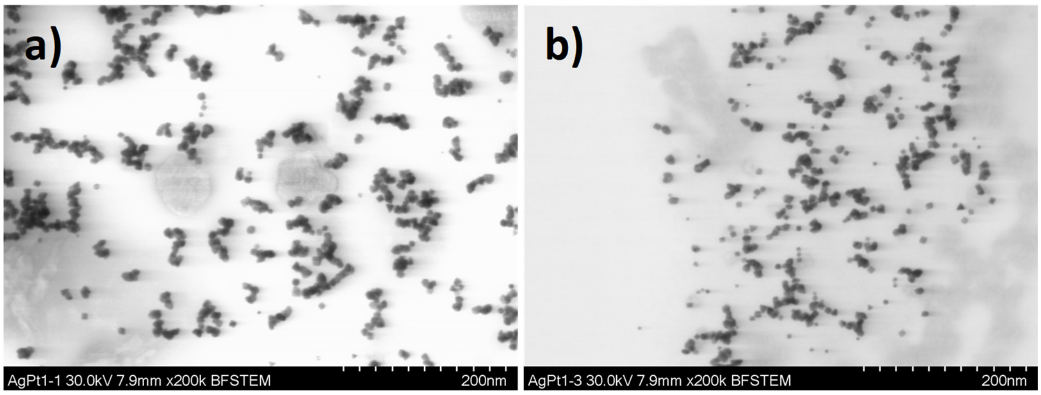

Figure S2. Low-magnification BF-STEM micrographs of a) AgPt (1:1) and b) AgPt (1:3) BNPs.

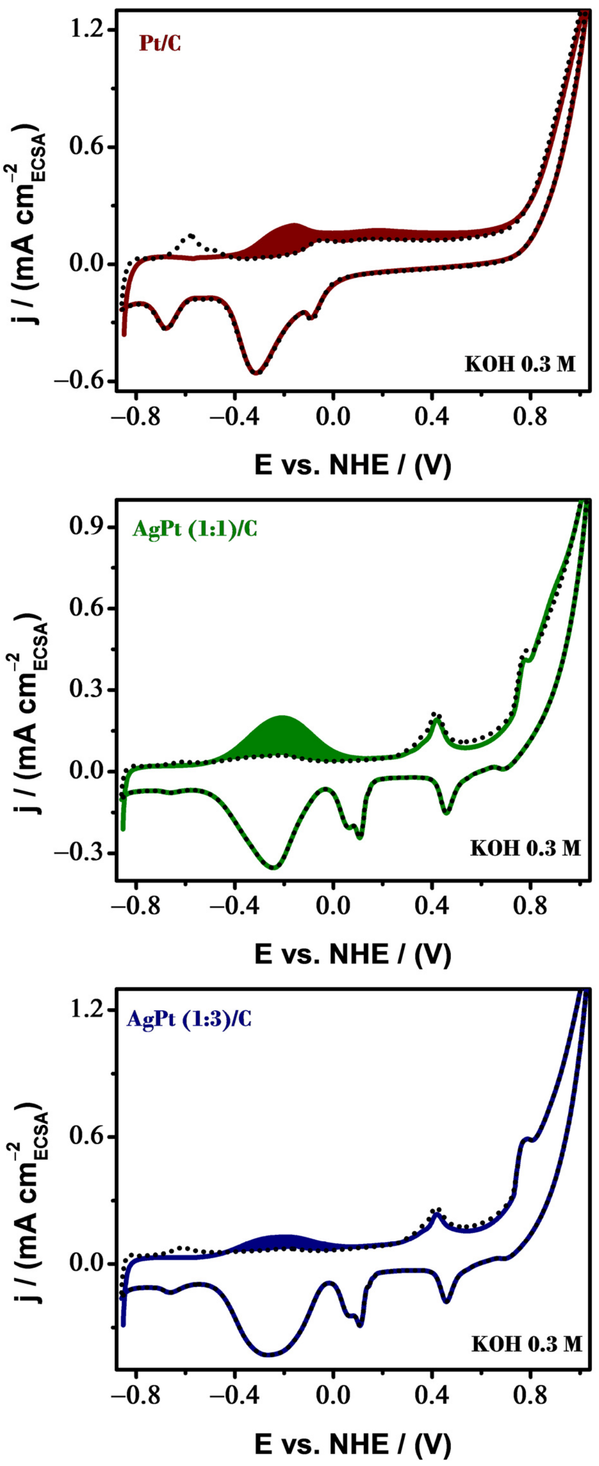

Figure S3. Cyclic voltammograms in alkaline media recorded consecutively after chronoamperometry (CA) measurements for Pt/C, AgPt (1:1)/C, and AgPt (1:3)/C electrocatalysts.

Table S1. Comparison of the catalytic activity of ethanol oxidation using Pt-based materials.

Material	Morphology/size	Test conditions	Catalytic activity	Reference
AgPt (1:3)/C	Sperical nanoparticles/10 nm	0.3 M KOH + 1.0 M Ethanol	3.17 mA cm^{-2}	This work
PtAg	Nanotubes/80 nm	1.0 NaOH + 1.0 M Ethanol	1.97 mA cm^{-2}	[1]
PtRu NWs/C	Nanowires/	0.1 M KOH +0.5 M Ethanol	3.78 mA cm^{-2}	[2]
PdPt/C	Nanoparticles/17 nm	0.3 M KOH + 1.0 M Ethanol	3.27 mA cm^{-2}	[3]
PtCu NCs/CB	Nanocubes/14 nm	1.0 M KOH + 1.0 M Ethanol	6.75 mA cm^{-2}	[4]
Pt_3Pd_2/C	Nanoparticles/3.4 nm	0.1 M NaOH + 0.1 M Ethanol	4.7 mA cm^{-2}	[5]
Pt_4Pd/C	Nanoparticles/3.5 nm	0.1 M NaOH + 0.1 M Ethanol	3.4 mA cm^{-2}	[5]
PtPbPd/C	Nanowires	0.5 M KOH + 0.5 M Ethanol	2.78 mA cm^{-2}	[6]
PtPd/C	Nanowires	0.5 M KOH + 0.5 M Ethanol	2.66 mA cm^{-2}	[6]
PtPb/C	Nanowires	0.5 M KOH + 0.5 M Ethanol	1.68 mA cm^{-2}	[6]

References

1. Gu, X.; Wang, C.; Yang, L.; Yang, C. 3d porous ptag nanotubes for ethanol electro-oxidation. *Journal of Nanoscience and Nanotechnology* **2017**, *17*, 2843-2847.
2. Yin, K.; Chao, Y.; Zeng, L.; Li, M.; Liu, F.; Guo, S.; Li, H. Ultrathin ptru nanowires as efficient and stable electrocatalyst for liquid fuel oxidation reactions. *Energy Mater. Adv.* **2022**, *2022*.
3. Higareda, A.; Mares-Briones, F.; Rosas, G.; Pérez, R.; Esparza, R. Enhanced durability of pdpt/c electrocatalyst during the ethanol oxidation reaction in alkaline media. *J. Solid State Electrochem.* **2022**, *26*, 2143-2151.
4. Liu, D.; Huang, H.-Z.; Zhu, Z.; Li, J.; Chen, L.-W.; Jing, X.-T.; Yin, A.-X. Promoting the electrocatalytic ethanol oxidation activity of pt by alloying with cu. *Catalysts* **2022**, *12*, 1562.
5. Jadali, S.; Kamyabi, M.A.; Solla-Gullón, J.; Herrero, E. Effect of pd on the electrocatalytic activity of pt towards oxidation of ethanol in alkaline solutions. *Appl. Sci.* **2021**, *11*, 1315.

6. Duan, J.-J.; Feng, J.-J.; Zhang, L.; Yuan, J.; Zhang, Q.-L.; Wang, A.-J. Facile one-pot aqueous fabrication of interconnected ultrathin ptpbpd nanowires as advanced electrocatalysts for ethanol oxidation and oxygen reduction reactions. *Int. J. Hydrog.* **2019**, *44*, 27455-27464.
